# The Mediating Effect of Inflammatory Biomarkers in the Associations Between Sarcoidosis and Incident Ischemic Stroke: A Prospective Cohort Study

**DOI:** 10.1002/brb3.71350

**Published:** 2026-03-26

**Authors:** Zihong Bai, Wenming Shao, Xiaxuan Huang, Shanyuan Tan, Yitong Ling, Shiqi Yuan, Xinya Li, Jun Lyu

**Affiliations:** ^1^ Department of Neurology The First Affiliated Hospital of Jinan University Guangzhou China; ^2^ Department of Emergency Medicine The First Affiliated Hospital of Jinan University Guangzhou China; ^3^ Department of Anesthesiology The First Affiliated Hospital of Jinan University Guangzhou China; ^4^ Department of Neurology The Second People's Hospital of Guiyang (The Affiliated Jinyang Hospital of Guizhou Medical University) Guiyang Guizhou China; ^5^ School of Nursing Jinan University Guangzhou China; ^6^ Department of Clinical Research the First Affiliated Hospital of Jinan University Guangzhou China; ^7^ Key Laboratory of Regenerative Medicine of Ministry of Education Guangzhou Guangdong China

**Keywords:** GEO, inflammatory, ischemic stroke, sarcoidosis, UK Biobank

## Abstract

**Background and objective:**

Sarcoidosis, characterized by granulomatous inflammation across multiple systems, has an unclear connection with ischemic stroke and shared molecular pathways. This study explores the link between sarcoidosis and ischemic stroke, focusing on the role of inflammatory markers using large population datasets and multi‐omics approaches.

**Methods:**

Data from 452,382 UK Biobank participants, followed for an average of 13.8 years, were combined with transcriptomic profiles from the GEO database (GSE58294 and GSE19314). A multivariable Cox regression was used to assess the association between sarcoidosis and ischemic stroke. Mediation analysis evaluated the role of inflammatory markers. Differential gene expression, GO and KEGG pathway enrichment, and protein–protein interaction networks were analyzed to explore shared molecular frameworks.

**Results:**

Sarcoidosis (*n* = 1760 cases) was found to independently increase the risk of ischemic stroke (HR = 1.335, 95% CI: 1.002–1.779). Sensitivity analysis using propensity score matching yielded consistent effect estimates (HR = 1.37). Mediation analysis identified the systemic immune‐inflammation index (SII, 8.43%), lymphocyte percentage (7.38%), and C‐reactive protein (CRP, 2.17%) as significant intermediaries. Transcriptomic analysis identified three genes—ANKRD22, FCGR1A, and NOG—with differential expression in both conditions, highlighting the TGF‐β/BMP signaling pathway and immune regulatory network as shared molecular underpinnings.

**Conclusion:**

This study provides the first evidence of a significant association between sarcoidosis and ischemic stroke in a large cohort. By integrating analytical methods, it highlights the role of inflammatory factors and potential molecular interactions between immune and developmental pathways, offering new insights for stroke risk reduction in sarcoidosis patients.

## Graphical abstract

Can invasive suckermouth catfish, *Hypostomus plecostomus*, be used as fishmeal? A potential solution to eradicate it from the Buriganga River, Bangladesh

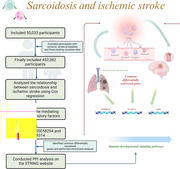

## 1 Introduction

Stroke is the second leading cause of mortality worldwide and a major contributor to disability, imposing a substantial burden on patients, families, and healthcare systems due to its high incidence, mortality, and disability rates. Among stroke subtypes, ischemic stroke (cerebral infarction) is the most prevalent, accounting for 55% to 80% of all cases globally. Therefore, early identification and mitigation of associated risk factors are essential in reducing its incidence (Steinmetz et al. 2024).

Sarcoidosis is a systemic granulomatous disease of unknown etiology (Drent et al. 2021). Epidemiological studies estimate its incidence at 1 to 15 cases per 100,000 individuals, with the highest prevalence observed in Northern Europe (11–15 per 100,000) and the lowest in East Asia (0.5–1 per 100,000) (Rossides et al. 2023). Furthermore, approximately 70% of cases occur between the ages of 25 and 40, with a secondary peak in women over 50 years old (Sève et al. 2021). Sarcoidosis can affect multiple organs, most commonly the lungs, eyes, and skin. Pulmonary involvement, including mediastinal lymphadenopathy and parenchymal disease, is the most frequent manifestation, occurring in approximately 90% of cases. Current knowledge of its pathogenesis primarily derives from research on pulmonary sarcoidosis (Bradshaw et al. 2021). The standard treatment for sarcoidosis involves immunotherapy, with corticosteroids recommended when clinical indications necessitate intervention (Brennan and Breen 2022). Recent studies suggest that novel therapeutics targeting CD4 type 1 helper T cell–mediated pathways may offer enhanced efficacy (Iannuzzi 2011).

Retrospective cohort studies have reported an increased incidence of cardiovascular diseases among patients with sarcoidosis (Ungprasert et al. 2017a; Ungprasert et al. 2017b). The pathogenesis of ischemic stroke is driven by a complex interaction between traditional vascular risk factors, such as atherosclerosis (Zheng et al. 2023), and systemic inflammatory diseases (Yu et al. 2023). Sarcoidosis is characterized by chronic activation of macrophages and T lymphocytes, leading to excessive secretion of pro‐inflammatory cytokines, including TNF‐α and CRP (Amber et al. 2015). These cytokines contribute to endothelial dysfunction, platelet activation, and cerebral microvascular injury—key mechanisms in stroke pathophysiology (cang et al. 2019; Chaturvedi and De Marchis 2024; Kumari et al. 2024). This suggests a potential shared inflammatory pathway between sarcoidosis and ischemic stroke. However, the precise molecular mechanisms linking these conditions remain inadequately understood, particularly regarding the potential mediating role of inflammatory markers.

This study aims to investigate the association and shared molecular pathways between sarcoidosis and ischemic stroke using prospective data from the UK Biobank and high‐throughput sequencing datasets from the Gene Expression Omnibus (GEO). Additionally, the study seeks to explore the potential mediating role of inflammatory markers in this relationship.

## 2 Materials and Methods

### 2.1 Study Population

The study population for this prospective cohort analysis was drawn from the UK Biobank (UKB), which enrolled over 500,000 participants aged 40–69 years between 2006 and 2010. All participants completed baseline questionnaires, provided biological samples, and underwent physical examinations at one of 22 assessment centers across England, Wales, and Scotland. The UK Biobank operates under ethical approval from the Multicenter Research Ethics Committee (REC reference: 11/NW/08320), and written informed consent was obtained from all participants (Sudlow et al. 2015). This study was performed under application number 104830. Individuals with a prior history of ischemic stroke at baseline and those with missing covariate data (*n* = 50,033) were excluded, yielding a final cohort of 452,382 participants.

**FIGURE 1:**
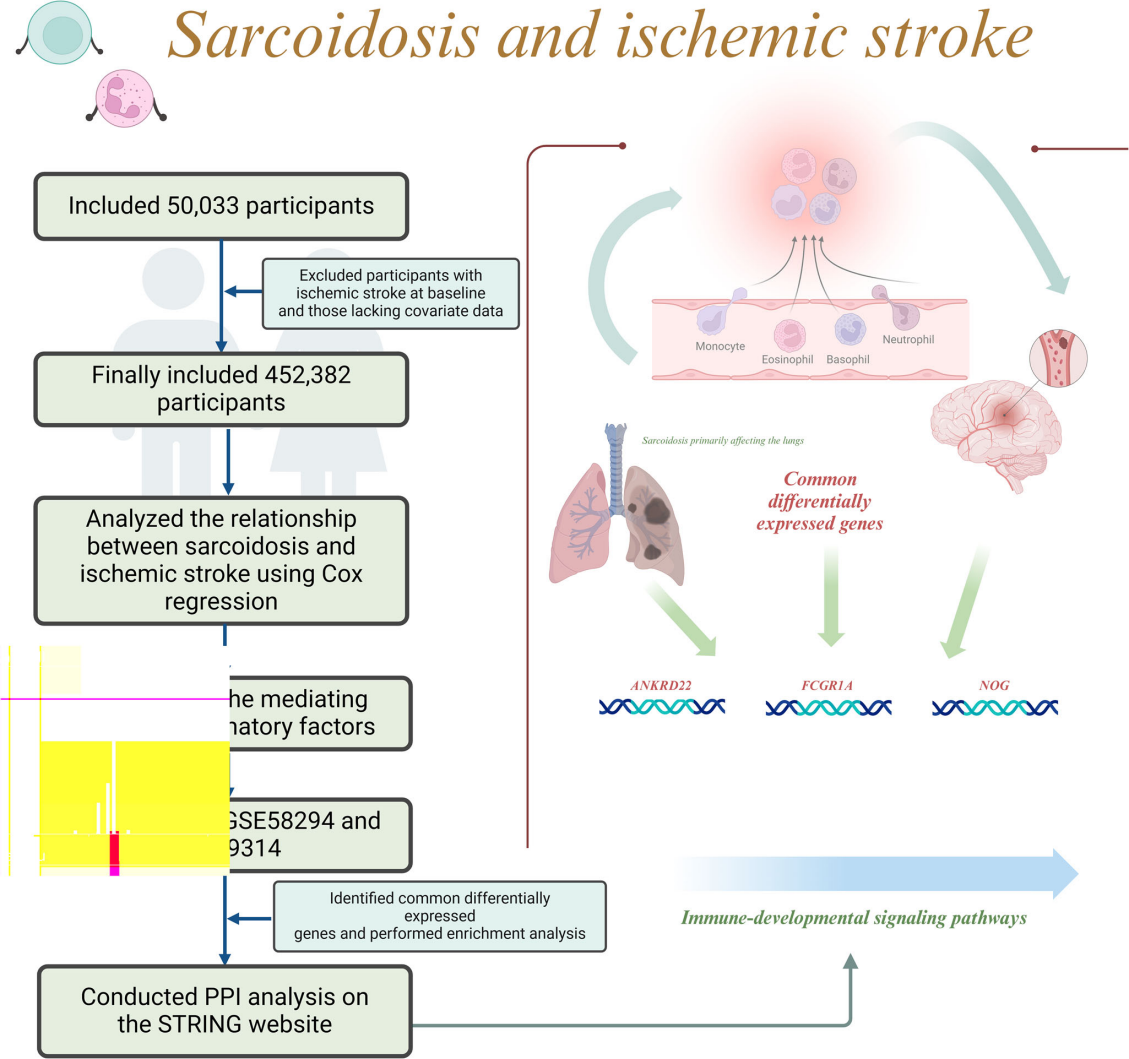
Mechanistic pathway.

Expression datasets for ischemic stroke, sarcoidosis, and normal samples were retrieved from the GEO database (http://www.ncbi.nlm.nih.gov/geo/). The study workflow is depicted in Figure 1 (created with BioRender). The inclusion criteria were as follows: (1) utilization of the GPL570 [HG‐U133_Plus_2] Affymetrix Human Genome U133 Plus 2.0 Array for all datasets; (2) human subjects exclusively; (3) a minimum of 20 samples per study; (4) samples derived from peripheral whole blood transcriptome analyses. Ultimately, datasets GSE58294 and GSE19314 were selected based on data reliability and extensive use in published literature (Stamova et al. 2014; Su et al. 2014). The GSE58294 dataset comprised 23 healthy individuals and 69 patients with cardioembolic stroke, whereas the GSE19314 dataset included 37 sarcoidosis patient samples, 20 controls without inflammatory diseases, and 6 patients with hypersensitivity pneumonitis.

### 2.2 Ischemic Stroke

Incident ischemic stroke was identified from hospital admission records in England, Scotland, and Wales using the International Classification of Diseases (ICD‐10) code I63.

### 2.3 Sarcoidosis

Incident sarcoidosis was determined from hospital admission records in England, Scotland, and Wales using the ICD‐10 code D86.

### 2.4 Inflammatory Indices

To assess the mediating role of inflammatory markers between sarcoidosis and ischemic stroke, specific immune indices reflecting various inflammatory processes were selected. These indices included C‐reactive protein (CRP) levels (mg/L), lymphocyte percentage, neutrophil‐to‐lymphocyte ratio (NLR), lymphocyte‐to‐monocyte ratio (LMR), platelet‐to‐lymphocyte ratio (PLR), and the systemic immune‐inflammation index (SII, defined as the product of the platelet count and the ratio of neutrophils to lymphocytes) (Ma et al. 2023).

### 2.5 Covariates

Demographic variables comprised age, sex, ethnicity, education level, and the Townsend Deprivation Index at baseline. The Townsend Deprivation Index, derived from census data on car ownership, overcrowding, home ownership, and unemployment, is a region‐based socioeconomic measure expressed as either positive or negative values, with positive values indicating greater deprivation (Cen et al. 2024). Lifestyle factors included smoking status (former, current, never), alcohol consumption (former, current, never), and self‐rated overall health. Self‐rated overall health was assessed as a subjective measure using a standard touchscreen questionnaire. Participants were asked, “In general how would you rate your overall health?” and selected one of four options: “Excellent,” “Good,” “Fair,” or “Poor.” Responses of “Do not know” or “Prefer not to answer” were treated as missing values. Comorbid conditions were defined by the presence of diabetes and hypertension (yes/no). Metabolic parameters included triglycerides (mmol/L), high‐density lipoprotein cholesterol (mmol/L), low‐density lipoprotein cholesterol (mmol/L), and body mass index (BMI).

### 2.6 Statistical Analysis

#### 2.6.1 UK Biobank Dataset

Categorical variables were summarized as counts (percentages), whereas continuous variables were presented as means ± standard deviations for normally distributed data or as interquartile ranges for non‐normally distributed data. The association between sarcoidosis and the risk of ischemic stroke was examined using a multivariable Cox proportional hazards regression model, with hazard ratios (HR) and confidence intervals (CI) estimated accordingly. The outcome was defined as the occurrence of ischemic stroke, with follow‐up censored at the date of stroke onset or at the last follow‐up (July 16, 2023). Sarcoidosis was treated as a binary exposure variable. Model 1 was unadjusted; Model 2 adjusted for age, sex, ethnicity, and the Townsend Deprivation Index; Model 3 further included adjustments for smoking status, alcohol consumption, overall health rating, BMI, diabetes, hypertension, triglycerides, high‐density lipoprotein, and low‐density lipoprotein (Yuan et al. 2023).

Model assumptions were evaluated using the Schoenfeld residuals test, and stratification methods were employed for variables that violated the proportional hazards assumption. Additionally, a Weibull accelerated failure time model was applied to validate the results, with HR estimates derived via exponential transformation of the scale parameter.

A dual regression model was constructed using the R package “mediation” (version 4.5.0) to investigate the mediating role of inflammatory markers in the relationship between sarcoidosis and ischemic stroke. The average causal mediation effect (ACME) was estimated via a nonparametric bootstrap procedure with 5000 resampling iterations. The inflammatory markers analyzed included CRP levels, lymphocyte percentage, NLR, LMR, PLR, and SII, with adjustments for age, sex, ethnicity, the Townsend Deprivation Index, smoking status, alcohol consumption, overall health rating, BMI, diabetes, hypertension, triglycerides, high‐density lipoprotein, and low‐density lipoprotein.

### 2.7 Sensitivity Analysis

To minimize selection bias and potential confounding due to baseline differences between the sarcoidosis and control groups, we performed a propensity score matching (PSM) analysis. We used a 1:4 nearest‐neighbor matching algorithm with a caliper width of 0.2 standard deviations. The propensity score was calculated using logistic regression based on all baseline covariates included in Model 3. Covariate balance before and after matching was assessed using the standardized mean difference (SMD), with an SMD < 0.1 considered indicative of good balance. Cox proportional hazards regression was then repeated in the matched cohort.

#### 2.7.1 GEO Database Dataset

##### 2.7.1.1 Differential Expression Analysis

Probe IDs from the GSE19314 and GSE58294 datasets were converted to official gene symbols. In cases where multiple probes mapped to the same gene, the probe exhibiting the highest expression level was selected. Differential expression analysis was performed independently on each dataset using the R package “limma” (version 3.62.2). A generalized linear model was employed to identify differentially expressed genes (DEGs) between diseased and control groups. DEGs were defined by a *p*‐value < 0.05, an absolute log2 fold change (|log2FC|) ≥ 0.5, and a false‐discovery rate (FDR) < 0.05. Genes meeting these criteria in both datasets were identified as common DEGs.

##### 2.7.1.2 Functional Enrichment Analysis

To elucidate the biological functions and pathways associated with the common DEGs, functional enrichment analyses were conducted. Gene Ontology (GO) and Kyoto Encyclopedia of Genes and Genomes (KEGG) pathway analyses were performed using the R package “clusterProfiler” (version 4.14.4). GO analysis included the categories of biological process (BP), molecular function (MF), and cellular component (CC). The entire genome annotation served as the background gene set, and enrichment significance was determined using hypergeometric testing. *p* Values were adjusted via the Benjamini–Hochberg method to obtain the FDR, with enriched categories filtered at an FDR < 0.05.

To further investigate the functional interactions among DEGs, a protein–protein interaction (PPI) network was constructed. Shared DEGs were uploaded to the STRING database (https://string‐db.org/) for analysis, with a confidence threshold set at 0.4 to identify biologically relevant interactions. STRING's functional enrichment tools were also utilized to assess network topology and perform GO and KEGG analyses, with significance determined by FDR correction (*p* < 0.05).

All analyses were performed using R (version 4.4.2), with statistical significance defined as *p* < 0.05.

## 3 Results

### 3.1 UK Biobank Database

#### 3.1.1 Population Characteristics

The participant selection process is detailed as follows. The initial UK Biobank cohort comprised 502,415 participants. After excluding 50,033 individuals due to prior history of ischemic stroke at baseline or missing covariate data, a total of 452,382 participants were included in the final analysis. Among them, 1760 were identified as incident sarcoidosis cases, while the remaining participants served as controls. Over a mean follow‐up of 13.8 years, 8218 participants experienced ischemic stroke, with females representing 40.3% and a mean (SD) age of 61.5 (6.55) years at the event. Notably, the prevalence of hypertension was significantly elevated among stroke cases compared with non‐cases. The baseline characteristics of the study population are summarized in Table 1.

**TABLE 1:** Baseline characteristics of the study population.

Characteristics	Total	Controls	Cases	*p* trend
*N*	452,382	444,164	8218	
Sex, *n* (%)				<0.001
Female	246,223 (54.4%)	242,908 (54.7%)	3315 (40.3%)	
Male	206,159 (45.6%)	201,256 (45.3%)	4903 (59.7%)	
Age, years, mean (SD)	56.5 (8.08)	56.4 (8.08)	61.5 (6.55)	<0.001
Race, *n* (%)				<0.001
White	412,761 (91.2%)	405,156 (91.2%)	7605 (92.5%)	
Other	39,621 (8.8%)	39,008 (8.8%)	613 (7.5%)	
Health status, *n* (%)				<0.001
Excellent	75,694 (16.7%)	7605 (92.5%)	848 (10.3%)	
Good	263,741 (58.3%)	259,429 (58.4%)	4312 (52.5%)	
Fair	93,671 (20.7%)	91,358 (20.6%)	2313 (28.1%)	
Poor	19,276 (4.26%)	18,531 (4.17%)	745 (9.07%)	
Smoking status, *n* (%)				<0.001
Never	248,044 (54.8%)	244,340 (55.0%)	3704 (45.1%)	
Previous	157,301 (34.8%)	154,030 (34.7%)	3271 (39.8%)	
Current	47,037 (10.4%)	45,794 (10.3%)	1243 (15.1%)	
Drinking status, *n* (%)				<0.001
Never	18,981 (4.20%)	18,549 (4.18%)	432 (5.26%)	
Previous	15,884 (3.51%)	15,486 (3.49%)	398 (4.84%)	
Current	417,517 (92.3%)	410,129 (92.3%)	7388 (89.9%)	
Education, *n* (%)				<0.001
College/university degree	148,996 (32.9%)	147,021 (33.1%)	1975 (24.0%)	
A/AS levels/equivalent	51,155 (11.3%)	50,417 (11.4%)	738 (8.98%)	
O levels/GCSEs/equivalent	97,145 (21.5%)	95,431 (21.5%)	1714 (20.9%)	
CSEs or equivalent	24,686 (5.46%)	24,363 (5.49%)	323 (3.93%)	
NVQ/HND/HNC/equivalent	29,948 (6.62%)	29,279 (6.59%)	669 (8.14%)	
Professional qualifications	23,718 (5.24%)	23,224 (5.23%)	494 (6.01%)	
None of the above	76734(16.98%) (5.24%)	74,429 (16.8%)	2305 (28.0%)	
Diabetes, *n* (%)				<0.001
No	429,440 (94.9%)	422,245 (95.1%)	7195 (87.6%)	
Yes	22,942 (5.07%)	21,919 (4.93%)	1023 (12.4%)	
Hypertension, *n* (%)				<0.001
No	272,030 (60.1%)	270,010 (60.8%)	2020 (24.6%)	
Yes	180,352 (39.9%)	174,154 (39.2%)	6198 (75.4%)	
Body mass index, kg/m^2^	27.4 (4.76)	27.4 (4.76)	28.4 (4.95)	<0.001
Townsend	−1.36 (3.06)	−1.36 (3.05)	−1.01 (3.22)	<0.001
Triglycerides, mmol/L	1.74 (1.03)	1.74 (1.02)	1.91 (1.09)	<0.001
Total cholesterol, mmol/L	5.70 (1.14)	5.70 (1.14)	5.55 (1.23)	<0.001
HDL, mmol/L	1.45 (0.38)	1.45 (0.38)	1.37 (0.38)	<0.001
LDL, mmol/L	3.56 (0.87)	3.56 (0.87)	3.48 (0.92)	<0.001

#### 3.1.2 Correlation Between Sarcoidosis and Ischemic Stroke

As indicated in Table 2, sarcoidosis was significantly associated with ischemic stroke across all three models (*p* < 0.05). In the fully adjusted Model 3, sarcoidosis remained significantly linked to an increased risk of ischemic stroke (HR = 1.335, 95% CI: 1.002–1.779), suggesting that individuals with sarcoidosis have a 1.335‐fold higher risk relative to those without the condition.

**TABLE 2:** Cox regression analysis.

Subgroup	Model 1*			Model 2*			Model 3*		
	*p* trend	HR	95% CI	*p* trend	HR	95% CI	*p* trend	HR	95% CI
Sarcoidosis **(yes/no)**	0.009	1.449	1.097–1.913	0.010	1.439	1.090–1.900	0.048	1.335	1.002–1.779

*Model 1: Unadjusted model. Model 2: Adjusted for sex, baseline age (years), race (White and others), and Townsend Deprivation Index. Model 3: Adjusted for the same variables as Model 2, as well as education level, smoking status (former smokers, current smokers, never smokers), alcohol consumption status (former drinkers, current drinkers, never drinkers), overall health rating (excellent, good, fair, poor), diabetes status (yes or no), hypertension status(yes or no), total cholesterol (mmol/L), total triglycerides (mmol/L), LDL (mmol/L), HDL (mmol/L).

**FIGURE 2:**
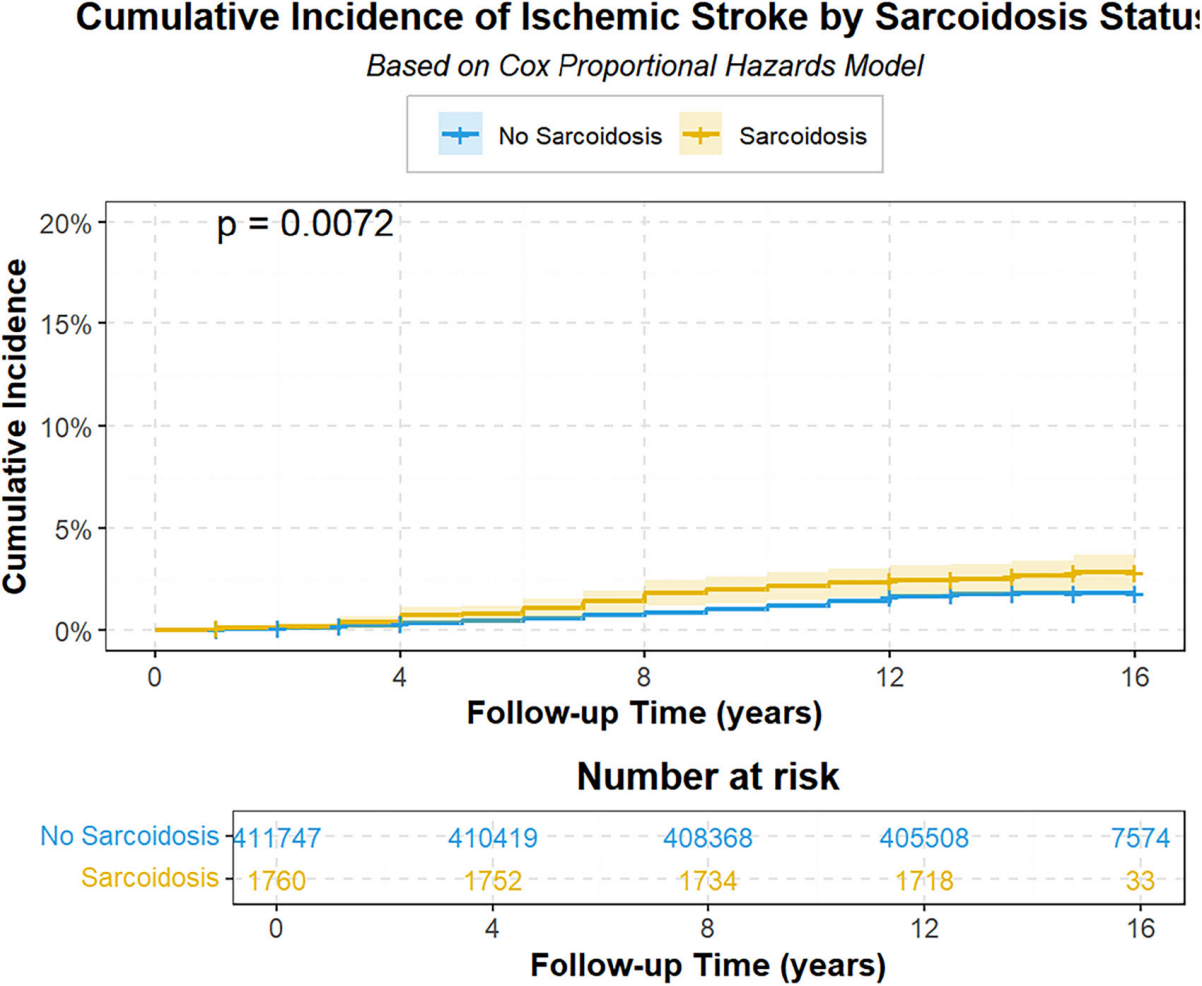
Cumulative incidence.

**FIGURE 3:**
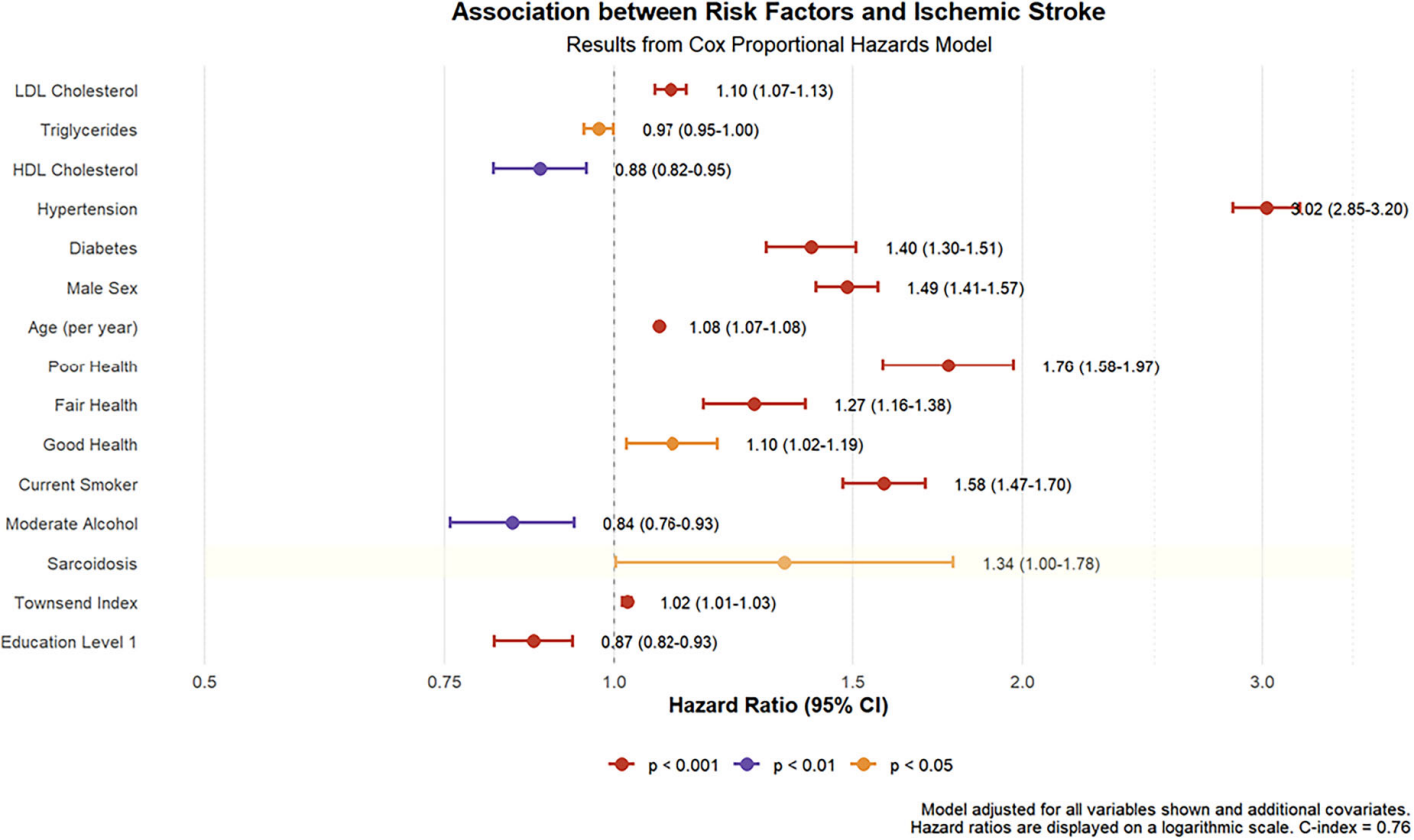
Multivariable‐adjusted hazard ratios.

Figure 2 illustrates a significant disparity in the cumulative incidence of ischemic stroke between subjects with and without sarcoidosis during follow‐up (*p* = 0.007), with sarcoidosis patients exhibiting a markedly elevated risk. Figure 3 presents a multivariable‐adjusted analysis based on Cox Model 3, revealing that the hazard ratio for sarcoidosis is comparable to that for smoking (HR = 1.58, 95% CI: 1.47–1.70) and diabetes (HR = 1.40, 95% CI: 1.30–1.51). Hypertension emerged as the strongest modifiable risk factor (HR = 3.02, 95% CI: 2.85–3.20), conferring a threefold increase in stroke risk compared to non‐hypertensive individuals. Additionally, moderate alcohol consumption, higher educational attainment, and elevated high‐density lipoprotein levels were protective, reducing ischemic stroke risk by 16%, 13%, and 12%, respectively. The forest plot further demonstrates an 8% increase in stroke risk per additional year of age.

Table 3 compares the original Cox multivariable model, the stratified Cox model, and the Weibull Cox model. The fully stratified model (stratified by sex, age, health, race, and smoking) passed the global test (*p* = 0.644), and the hazard ratio for the treatment variable remained stable over time (*p* = 0.106), thereby satisfying the proportional hazards assumption. The Weibull parametric model corroborated the direction (HR > 1) and magnitude (34% vs. 36.5%) observed in the Cox model, thus affirming the robustness of the risk effect associated with sarcoidosis.

**TABLE 3:** Sensitivity analyses for the association between sarcoidosis and ischemic stroke using different Cox models.

Method	HR (95% CI)	*p* value
Primary Cox model	1.335 (1.002–1.779)	0.048
Stratified Cox model (full stratification)	1.365 (1.023–1.821)	0.034
Weibull parametric model	1.34 (1.01–1.78)	0.048

*Note*: Primary Cox model:  Adjusted for all covariates listed in Model 3 of Table 2 (age, sex, ethnicity, Townsend Deprivation Index, education level, smoking status, alcohol consumption status, overall health rating, body mass index, diabetes, hypertension, triglycerides, HDL cholesterol, and LDL cholesterol). Stratified Cox model: Stratified by sex, age, health status, race, and smoking status; adjusted for the same covariates as the primary model. Weibull parametric model: Assuming a Weibull distribution for the survival time; adjusted for the same covariates as the primary model.

#### 3.1.3 Mediation Effects of Inflammatory Markers

Figure 4 demonstrates that the mediation effects of the systemic immune‐inflammation index (SII), lymphocyte percentage, and C‐reactive protein (CRP) were highly significant (*p* < 0.001), with mediation proportions of 8.43%, 7.38%, and 2.17%, respectively. The platelet‐to‐lymphocyte ratio (PLR) exhibited a significant mediation effect (*p* = 0.037) with a mediation proportion of 3.70%.

FIGURE 4Mediation analysis.

**Figure:**
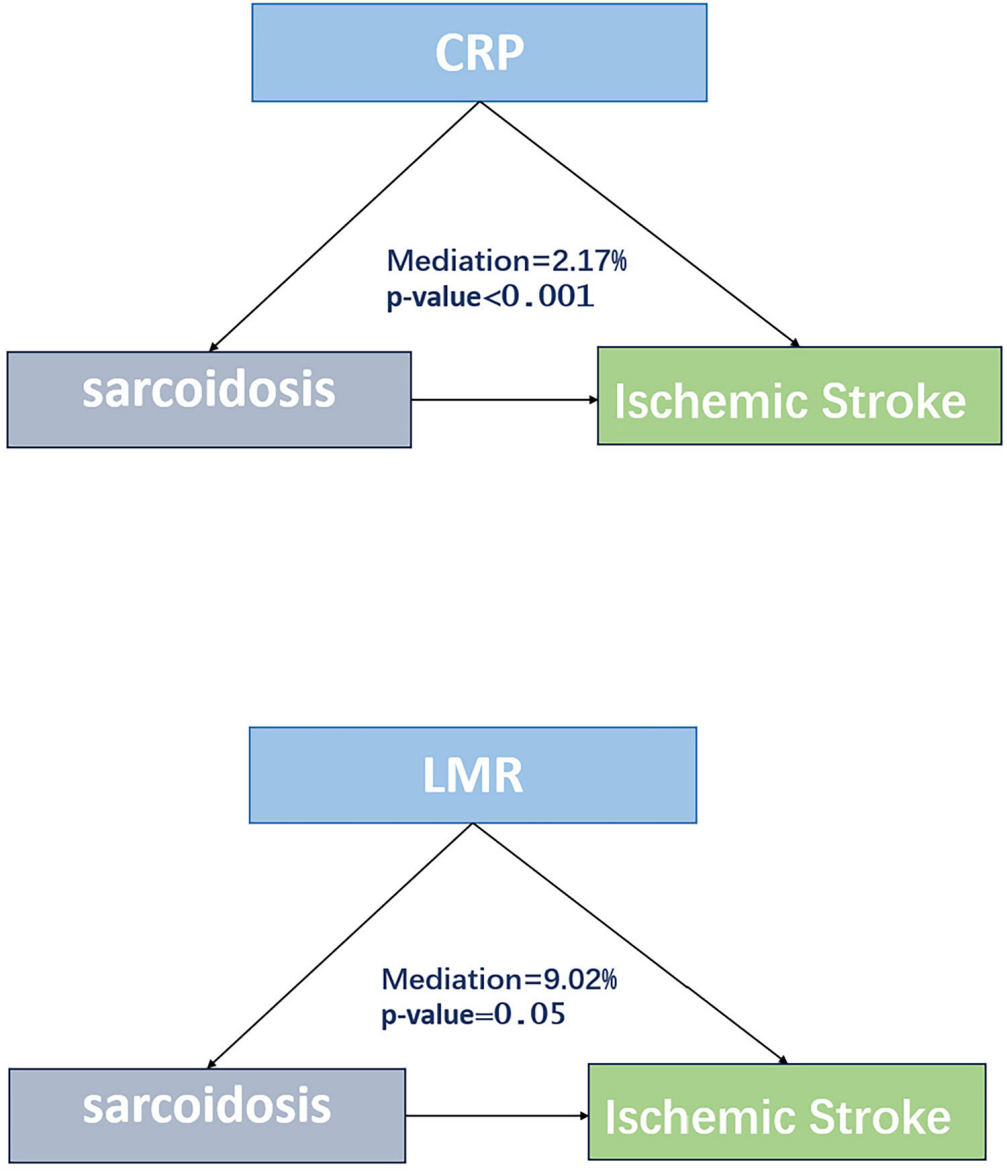

**Figure:**
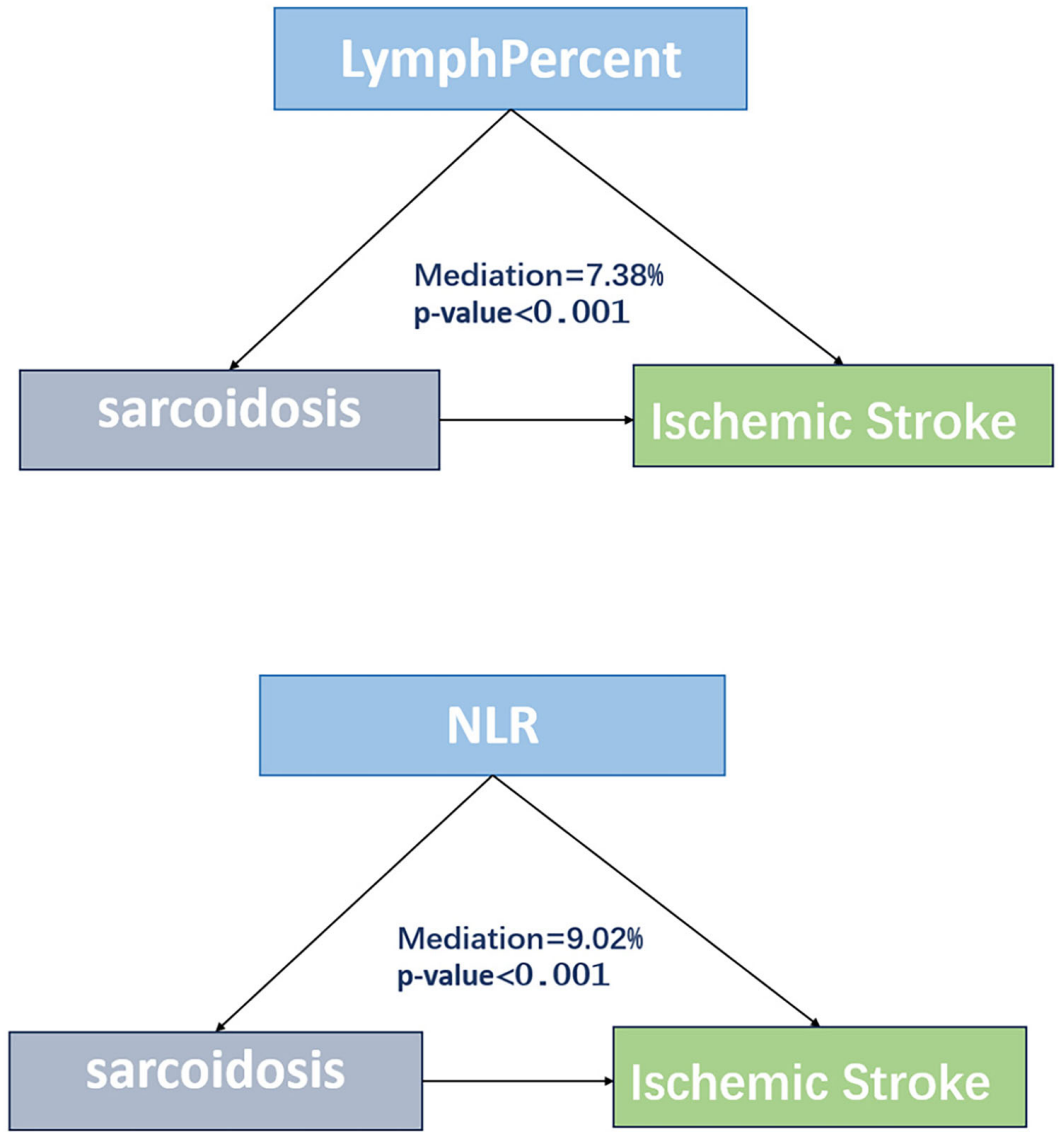

**Figure:**
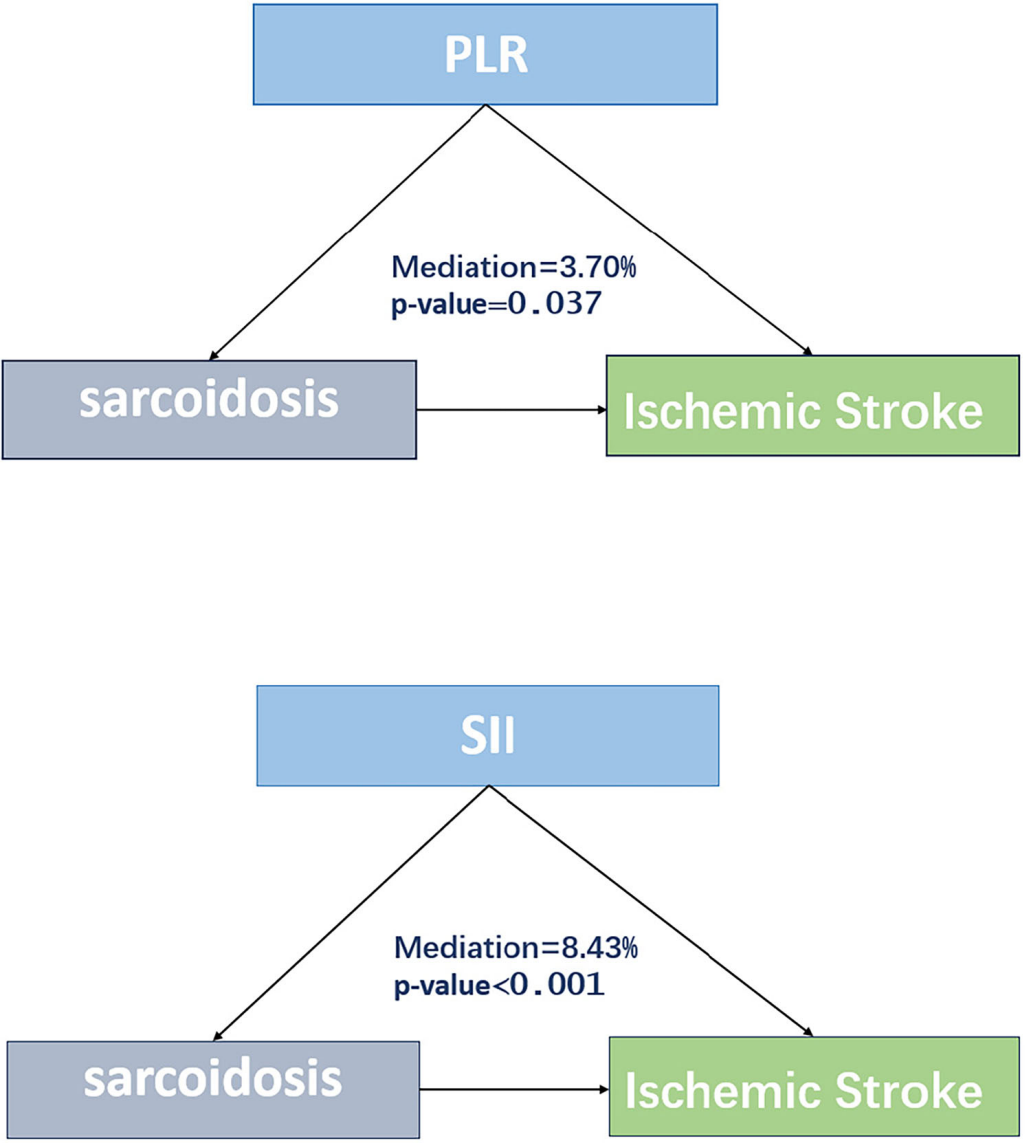

#### 3.1.4 Sensitivity Analysis Using Propensity Score Matching

To address potential confounding, a 1:4 propensity score matching was performed, resulting in a well‐balanced cohort of 1760 patients with sarcoidosis and 7040 controls (all standardized mean differences < 0.05; Table S1 and Figure S1).  In this matched cohort, the association between sarcoidosis and ischemic stroke was consistent with the primary analysis in terms of effect size, with a hazard ratio of 1.37 (95% CI: 0.98–1.91, *p* = 0.062).

### 3.2 GEO Database

#### 3.2.1 Selection of Differentially Expressed Genes

**FIGURE 5:**
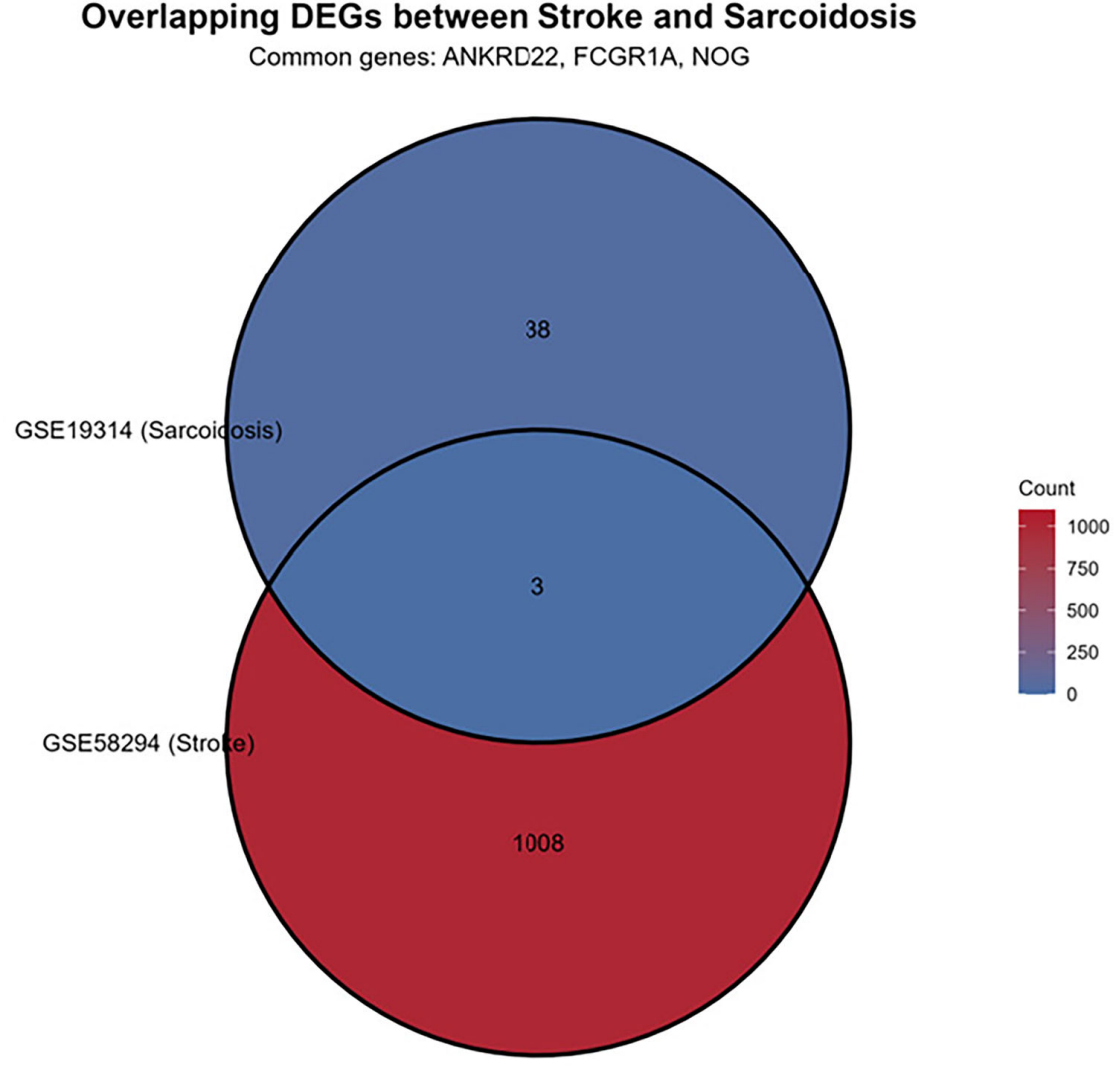
Venn.

Differential expression analysis was performed using the limma package on datasets GSE58294 and GSE19314. As depicted in Figure 5, the GSE58294 dataset yielded 1011 significantly differentially expressed genes, whereas the GSE19314 dataset for sarcoidosis produced 41 such genes. The intersection of these analyses identified three shared differentially expressed genes: ANKRD22, FCGR1A, and NOG (see Supplementary Material for the complete gene list).

#### 3.2.2 GO and KEGG Enrichment Analysis

**FIGURE 6:**
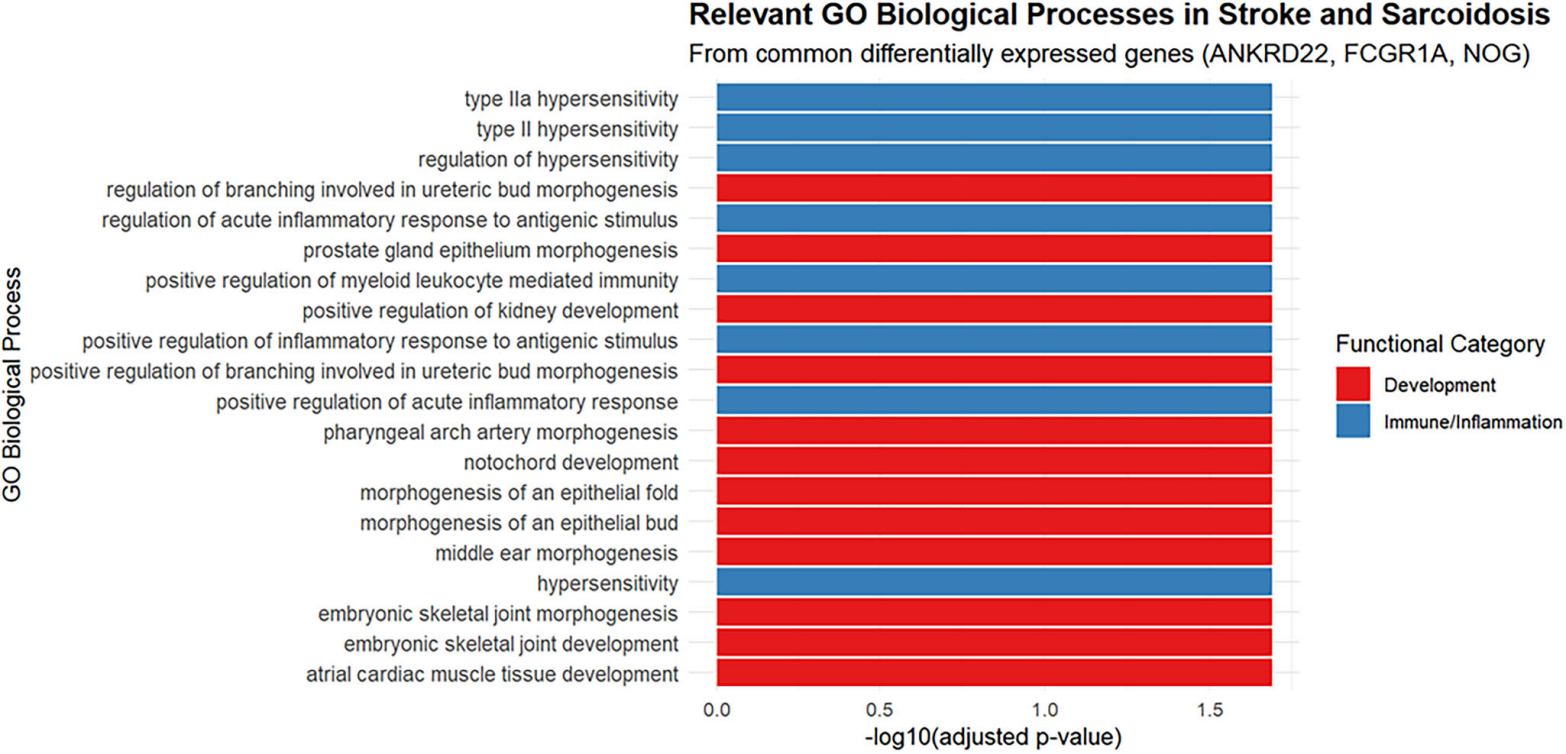
GO analysis.

**FIGURE 7:**
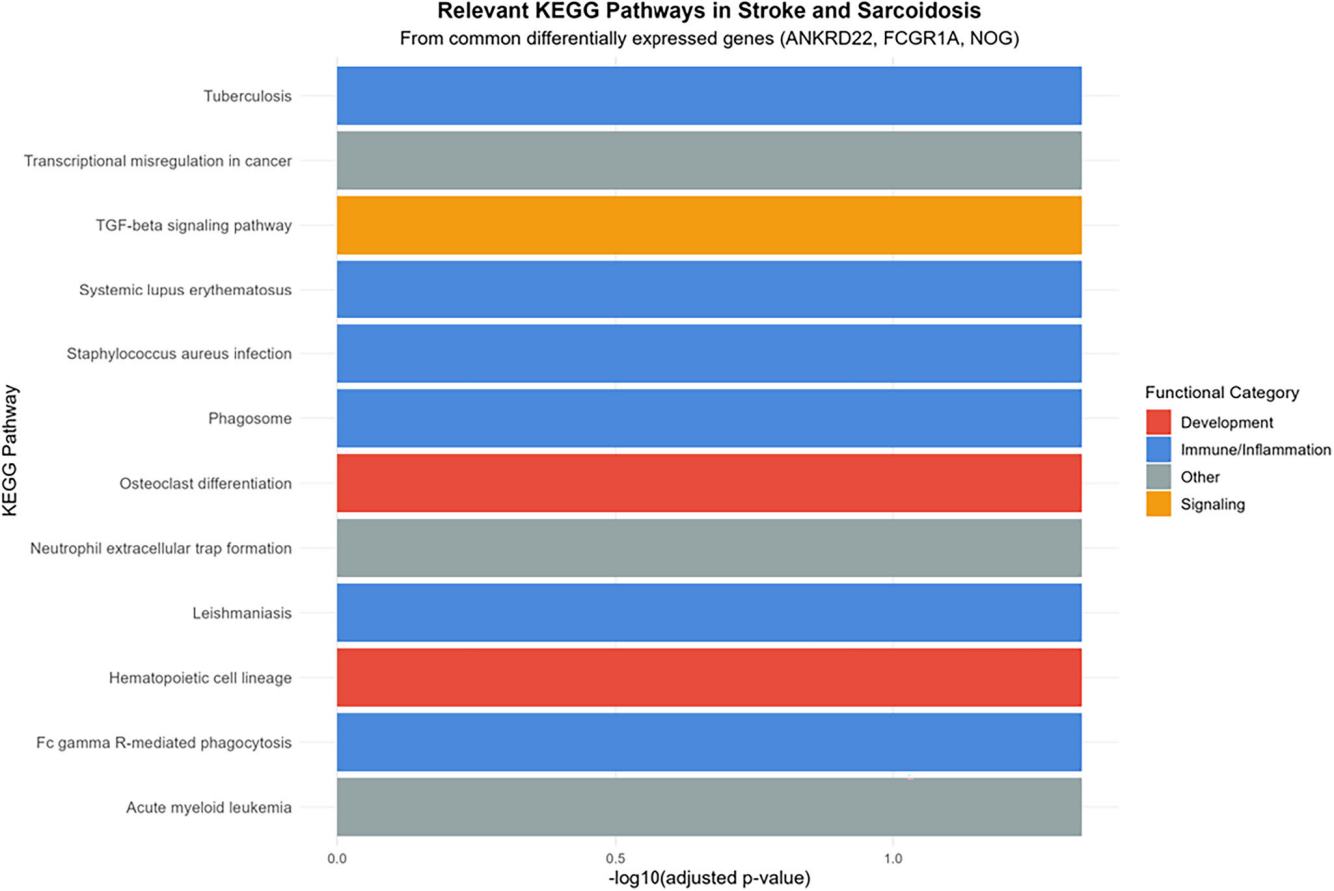
KEGG analysis.

To elucidate the biological processes and signaling pathways associated with these genes, Gene Ontology (GO) and Kyoto Encyclopedia of Genes and Genomes (KEGG) enrichment analyses were conducted. The GO analysis (Figure 6) revealed that ANKRD22, FCGR1A, and NOG were primarily enriched in two functional domains: regulation of immune responses, including type III hypersensitivity reactions, acute inflammatory response modulation, and myeloid leukocyte‐mediated immunity (blue section); and tissue injury repair and remodeling (red section). These results underscore the imbalance between inflammation‐induced tissue damage and subsequent reparative processes, which may constitute a common pathophysiological mechanism in both conditions. The KEGG analysis (Figure 7) identified shared molecular mechanisms between sarcoidosis and ischemic stroke, particularly involving immune regulation and tissue development pathways.

#### 3.2.3 Protein–Protein Interaction Network Analysis

**FIGURE 8:**
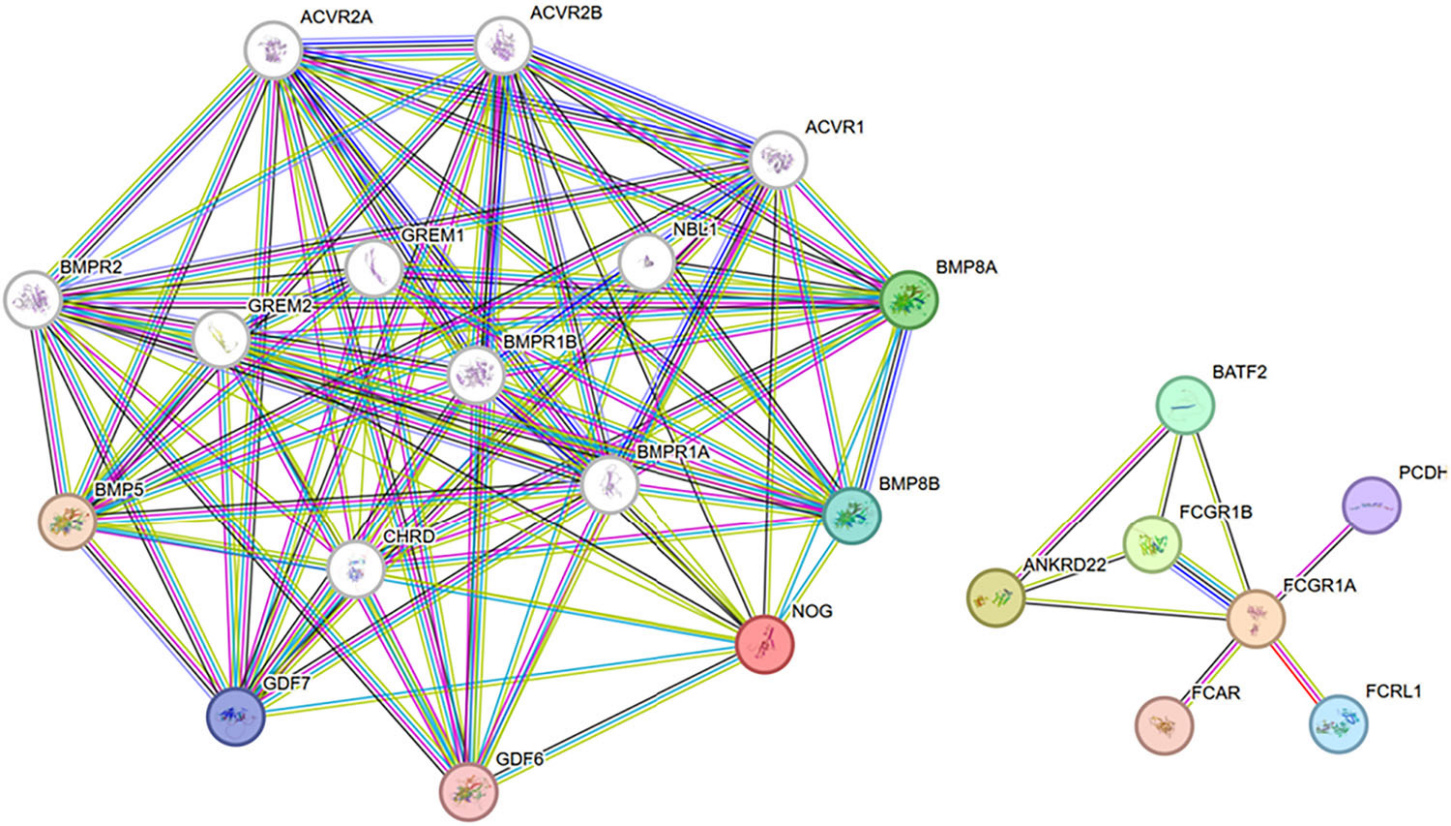
PPI.

A protein–protein interaction (PPI) network was constructed using the STRING database (v11.5), with the three shared differentially expressed genes serving as seed nodes and an interaction confidence threshold of 0.4. In the resultant network (Figure 8), nodes were color‐coded according to functional groups, and edges represented various types of interaction evidence. The network comprised 24 protein nodes and 78 interaction edges, which segregated into two distinct functional modules. The left module, centered on NOG, is indicative of a developmental signaling cluster involving 16 proteins primarily engaged in the BMP/TGF‐β superfamily signaling pathway, with key nodes including NOG, multiple BMP receptors (BMPR1A/1B/2), and activin receptors (ACVR1/2A/2B). In contrast, the right module, centered on FCGR1A/ANKRD22, represents an immune regulatory cluster containing eight proteins predominantly involved in immune response modulation and signal transduction. FCGR1A interacts with various Fc receptor‐related proteins (FCGR1B, FCRL1, FCAR), while ANKRD22 is posited to form a transcriptional regulatory axis with BATF2. These findings suggest a shared molecular mechanism underlying stroke and sarcoidosis, characterized by dysregulated immune responses and aberrant developmental signaling.

The GO analysis (Supplementary Material) revealed pronounced enrichment of the BMP signaling pathway (FDR ≈ 1.0e‐20) with a signal strength of approximately 6.5, alongside significant enrichment of SMAD protein phosphorylation regulatory pathways (FDR ≈ 1.0e‐18). The KEGG analysis (Supplementary Material) demonstrated extremely significant enrichment of the TGF‐β signaling pathway (FDR ≈ 1.0e‐29) with a signal strength of 9.1, as well as enrichment in the “fluid shear stress and atherosclerosis” pathway, thereby providing direct evidence linking the NOG‐BMP signaling axis to the vascular pathology observed in stroke.

## 4 Discussion

This study reveals a significant association between sarcoidosis and ischemic stroke. By integrating prospective cohort analysis from the UK Biobank with transcriptomic data from GEO, we uncovered epidemiological evidence and potential shared molecular mechanisms linking sarcoidosis and ischemic stroke. Additionally, inflammatory factors were found to play a crucial mediating role in this association.

Previous research has reported cases of sarcoidosis presenting as stroke (Brown et al. 1989), and subsequent single‐case or small cohort studies have analyzed sarcoidosis‐induced stroke, such as a cohort study from the Massachusetts General Brigham (MGB) Research Patient Data Registry describing cases of neurosarcoidosis with cerebrovascular complications primarily presenting as ischemia (Hutto et al. 2022). In our study, based on a prospective cohort of over 450,000 UK Biobank participants, we confirmed that sarcoidosis significantly increases the risk of ischemic stroke (HR = 1.50, 95% CI: 1.01–1.91), with this association remaining significant even after comprehensive adjustment for traditional vascular risk factors (HR = 1.335, 95% CI: 1.002–1.779). This finding aligns with previous observations that autoimmune diseases increase the risk of cardiovascular events (Ungprasert et al. 2017b). In this model, hypertension posed the highest risk (HR = 3.02, 95% CI: 2.85–3.20), underscoring the importance of blood pressure management in sarcoidosis patients. Furthermore, the risk associated with sarcoidosis was comparable to that of smoking (HR = 1.58) and diabetes (HR = 1.40), highlighting its clinical significance as a risk factor for ischemic stroke.

The robustness of this association was further evaluated through a sensitivity analysis using propensity score matching. Although the statistical significance was attenuated (*p* = 0.062) in the matched cohort—likely due to the reduced sample size—the effect estimate remained highly consistent (HR = 1.37), closely aligning with that from the primary multivariable‐adjusted model (HR = 1.34). This consistency in the point estimates, achieved after balancing a comprehensive set of baseline covariates, strongly suggests that the observed association is not substantially driven by residual confounding and enhances the credibility of our main results.

Through differential expression analysis of GSE58294 and GSE19314 datasets, we identified three key genes—ANKRD22, FCGR1A, and NOG—that are differentially expressed in both diseases. GO and KEGG pathway enrichment analyses revealed significant enrichment in two major biological processes: (1) Developmental Pathways: From tissue morphogenesis to organ repair. GO analysis highlighted significant enrichment in development‐related biological processes (e.g., ureteric bud branching, prostate epithelial morphogenesis, notochord development, embryonic skeletal joint development), all closely related to NOG‐mediated BMP/TGF‐β signaling regulation. As a BMP antagonist, NOG regulates tissue morphogenesis and organ repair by binding to BMP ligands and blocking their interaction with receptors (Bravo‐Nuevo et al. 2016; Gerhart et al. 2006). KEGG analysis further confirmed significant enrichment of the TGF‐β signaling pathway (highest signal strength of 9.1) and involvement of osteoclast differentiation and hematopoietic cell lineage pathways related to tissue development (Deng et al. 2024). The shared mechanism of these developmental pathways in stroke and sarcoidosis can be explained as follows: following tissue injury, dysregulation of the NOG‐BMP signaling axis leads to abnormal repair processes, manifesting as aberrant vascular remodeling and impaired neural repair in stroke, and granuloma formation and tissue fibrosis in sarcoidosis. Abnormal activation of this axis in both diseases may represent an inappropriate repair response to injury, ultimately leading to functional impairment. (2) Immune Regulatory Pathways: GO analysis showed significant enrichment in immune/inflammation‐related biological processes (e.g., type II and IIa hypersensitivity reactions, regulation of acute inflammatory responses, myeloid leukocyte‐mediated immunity). KEGG analysis revealed involvement of multiple immune‐related pathways, including tuberculosis, systemic lupus erythematosus, and *Staphylococcus aureus* infection. These findings underscore the central role of FCGR1A and ANKRD22 in immune regulation. FCGR1A, a high‐affinity IgG Fc receptor, plays a critical role in antibody‐mediated effector responses and has been identified as a risk factor for sarcoidosis (Wu et al. 2022). ANKRD22 is associated with immune responses (Chen et al. 2023). In sarcoidosis, abnormal immune activation leads to granuloma formation, while in ischemic stroke, excessive immune activation exacerbates neuroinflammation and secondary injury. The shared immune abnormalities in both diseases suggest similar inflammatory cascades and potential common therapeutic targets.

Protein–protein interaction network analysis also revealed two distinct but functionally interrelated modules, providing a molecular basis for the “immune‐developmental axis” hypothesis and validating the GO and KEGG analyses of the three differentially expressed genes. The developmental signaling module centered on NOG plays a critical role in tissue repair and remodeling. GO and KEGG analyses demonstrated significant enrichment of the BMP signaling pathway (FDR ≈ 1.0e‐20) and TGF‐β signaling pathway (FDR ≈ 1.0e‐29), not only emphasizing their association in both diseases but also suggesting potential molecular intervention targets. Additionally, enrichment of the “fluid shear stress and atherosclerosis” pathway directly links the NOG‐BMP signaling axis to stroke vascular pathology, consistent with previous reports on BMP signaling's involvement in vascular endothelial function and vascular wall remodeling (Ciais and Bailly 2012; Orlova et al. 2016). On the other hand, the immune regulatory module composed of FCGR1A and ANKRD22 represents a bridge between innate and adaptive immunity. This study is the first to propose a potential interactive regulatory relationship between these two functional modules, forming an “immune‐developmental axis” that drives disease progression. This new paradigm may explain the clinical observation of coexisting inflammatory responses and tissue remodeling in sarcoidosis and stroke.

Crucially, the transcriptomic signatures identified in our study provide a potential molecular basis for the systemic inflammatory biomarkers observed in the mediation analysis. FCGR1A (CD64) is a high‐affinity IgG Fc receptor prominently expressed on activated monocytes and neutrophils (Hübner et al. 2025). Its upregulation aligns with the predictive value of the Systemic Immune‐Inflammation Index (SII) (which incorporates neutrophil and platelet counts) and the Lymphocyte‐to‐Monocyte Ratio (LMR), suggesting that myeloid lineage activation is a key driver of the inflammatory milieu in sarcoidosis‐associated stroke. Furthermore, NOG acts as an antagonist to BMP signaling, a pathway essential for lymphocyte development and differentiation (Sconocchia and Sconocchia 2021). Dysregulation of the NOG‐BMP axis may impair lymphocyte homeostasis, mechanistically supporting our finding that lymphocyte percentage acts as a significant mediator. Thus, the observed gene expression changes (FCGR1A, NOG) likely manifest systemically as the alterations in blood cell ratios (SII, lymphocytes) that facilitate vascular pathology.

Furthermore, mediation analysis revealed that SII (8.43%), lymphocyte percentage (7.38%), and CRP (2.17%) play mediating roles in the association between sarcoidosis and ischemic stroke. These results support the molecular mechanisms mentioned above. The mediating effect of lymphocyte percentage may reflect the regulation of immune balance by the NOG‐BMP signaling pathway, as studies have shown that TGF‐β family members can modulate T‐cell differentiation and function (Chen 2023). Sarcoidosis is characterized by granulomatous inflammation involving macrophages and CD4 T cells (Saw and Song 2025), while patients with ischemic stroke exhibit elevated levels of inflammatory markers such as CRP, MCP‐1, and MPO (Cao et al. 2025). Our findings enhance our understanding of the role of inflammation in the association between sarcoidosis and ischemic stroke.

## 5 Limitations

This study has several limitations. First, despite using a large prospective cohort, the number of sarcoidosis cases was relatively small, and the population was predominantly White. Specifically, the propensity score matching analysis, while rigorous in controlling for confounding, further reduced the effective sample size, which may have limited the statistical power to detect significant associations in the sensitivity analysis. Although various potential confounders were controlled in the analysis, the observational nature of the study cannot exclude the possibility of other influences. Second, we acknowledge limitations regarding the mediation analysis. Information on sarcoidosis treatment (e.g., corticosteroids or immunosuppressants) was not fully available. Since these medications can alter inflammatory biomarkers (e.g., reducing lymphocyte counts or CRP levels), unmeasured confounding by medication use may have biased our mediation estimates. Additionally, inflammatory markers were measured only at baseline, reflecting a single time point rather than cumulative exposure. Third, the transcriptomic analysis was based on peripheral blood samples, which may not fully reflect tissue‐specific molecular changes. Fourth, the number of shared differentially expressed genes identified was limited, potentially failing to capture all shared molecular mechanisms. Future studies should, where possible, expand sample sizes and integrate transcriptomic data from multiple tissue types for comprehensive analysis.

## 6 Conclusion

In summary, by integrating epidemiological and molecular biological approaches, this study reveals the association between sarcoidosis and ischemic stroke and its underlying molecular mechanisms, emphasizing the importance of preventing ischemic stroke in patients with sarcoidosis.

## Author Contributions

**Zihong Bai: **Writing – original draft, Methodology. **Wenming Shao: **Methodology, Formal analysis, Writing – review & editing.** Xiaxuan Huang: **Investigation, Data curation.** Shanyuan Tan: **Methodology.** Yitong Ling: **Data curation. **Shiqi Yuan: **Validation. **Xinya Li: **Visualization. **Jun Lyu**: Supervision.

## Funding

The authors have nothing to report.

## Supporting information




**Supplementary Material**: brb371350‐sup‐0001‐figureS1.tiff

**Supplementary Material**: brb371350‐sup‐0001‐SuppMat.doc

**Supplementary Material**: brb371350‐sup‐0001‐tableS1.doc

## Data Availability

The dataset used in this study is publicly accessible through the UK Biobank (www.ukbiobank.ac.uk/).
