# Supplementary figures and images for: The Mediating Effect of Inflammatory Biomarkers in the Associations Between Sarcoidosis and Incident Ischemic Stroke: A Prospective Cohort Study
Source: Brain Behav. 2026 Mar 26;16(4):e71350. doi: 10.1002/brb3.71350 (PMC13109034; doi:10.1002/brb3.71350)

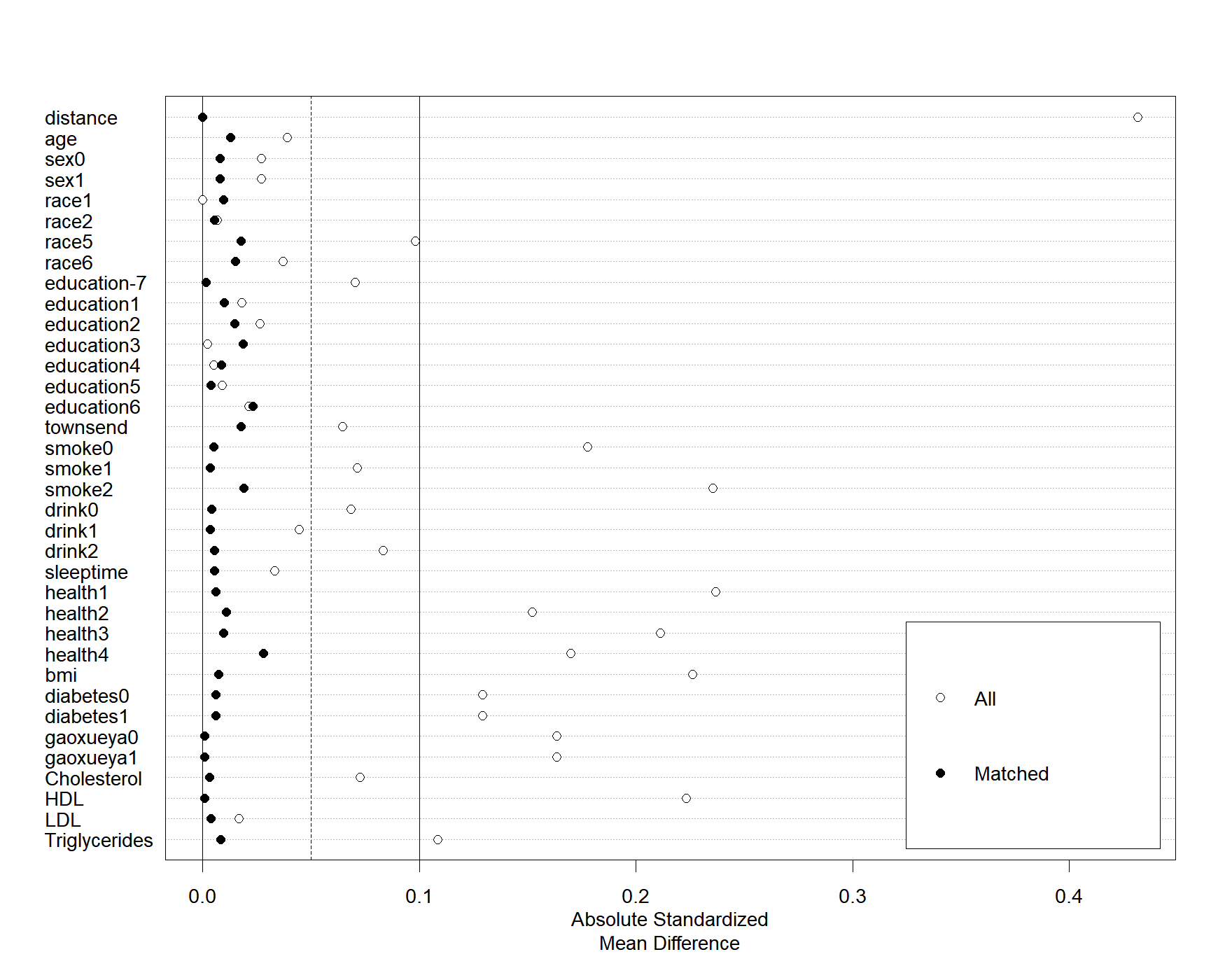

Supplement: Supplementary file 1 — Supplementary Material: brb371350‐sup‐0001‐figureS1.tiff [file BRB3-16-e71350-s002.tiff]
