# Supplementary material for: The Mediating Effect of Inflammatory Biomarkers in the Associations Between Sarcoidosis and Incident Ischemic Stroke: A Prospective Cohort Study
Source: Brain Behav. 2026 Mar 26;16(4):e71350. doi: 10.1002/brb3.71350 (PMC13109034; doi:10.1002/brb3.71350)

**Table**

1.Field ID in UK Biobank

| **Field ID Description** |  |
| --- | --- |
| sex | 31 |
| Date of attending assessment centre | 53 |
| Age when attended assessment centre | 21003 |
| Sleep duration | 1160 |
| Overall health rating | 2178 |
| Qualifications | 6138 |
| Smoking status | 20116 |
| Alcohol drinker status | 20117 |
| Ethnic background | 21000 |
| Townsend deprivation index at recruitment | 22189 |
| Body fat percentage | 23099 |
| Trunk fat percentage | 23127 |
| Date I63 first reported (cerebral infarction) | 131366 |
| Body mass index (BMI) | 21001 |
| Diastolic blood pressure, automated reading | 4079 |
| Systolic blood pressure, automated reading | 4080 |
| Date D86 first reported (sarcoidosis) | 130686 |
| LDL direct | 30780 |
| HDL cholesterol | 30760 |
| Triglycerides | 30870 |

2.common_gene_symbols

| GeneSymbol |
| --- |
| ANKRD22 |
| FCGR1A |
| NOG |

**Figure:**

1.Gene Ontology enrichment


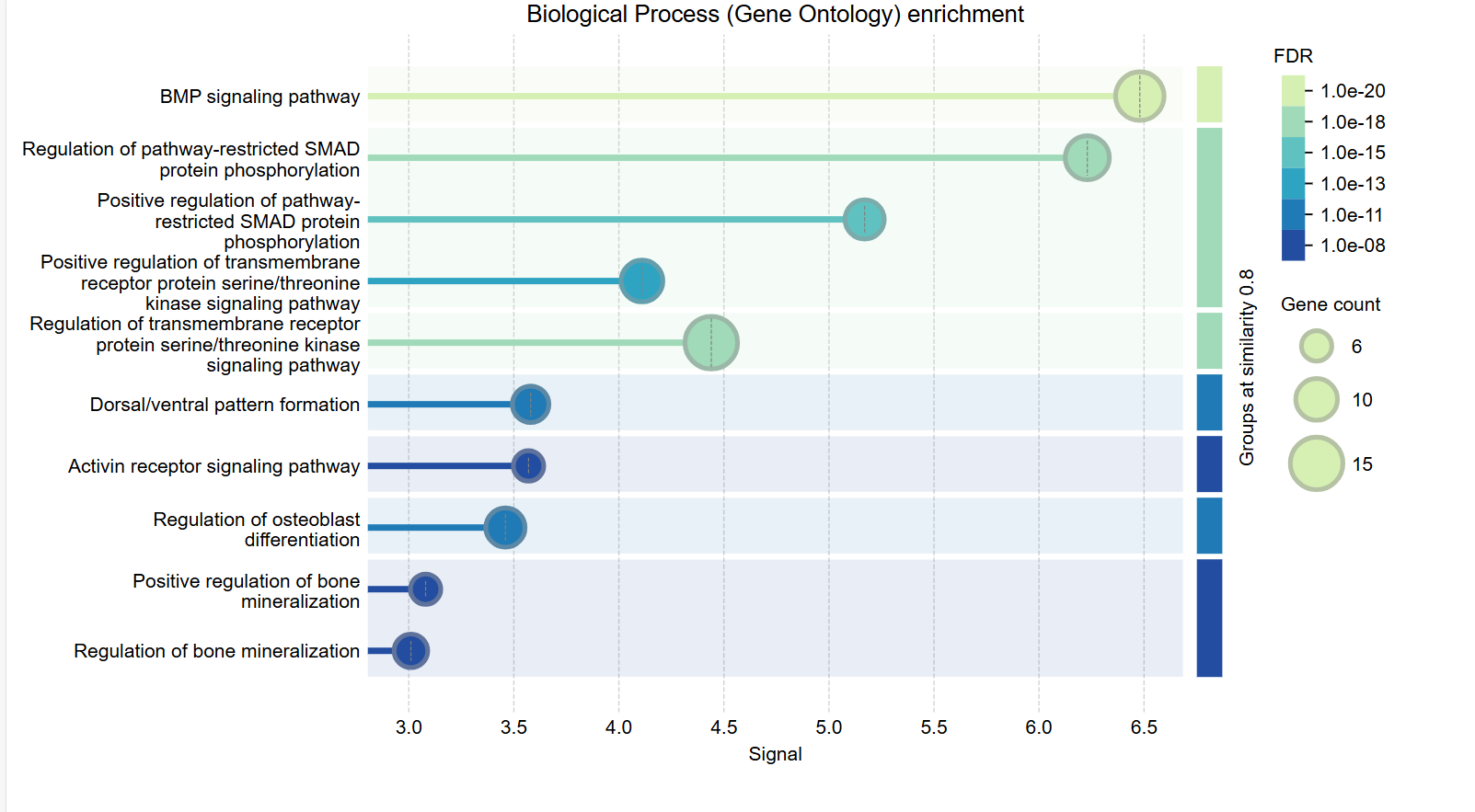


2.KEGG Pathways enrichment


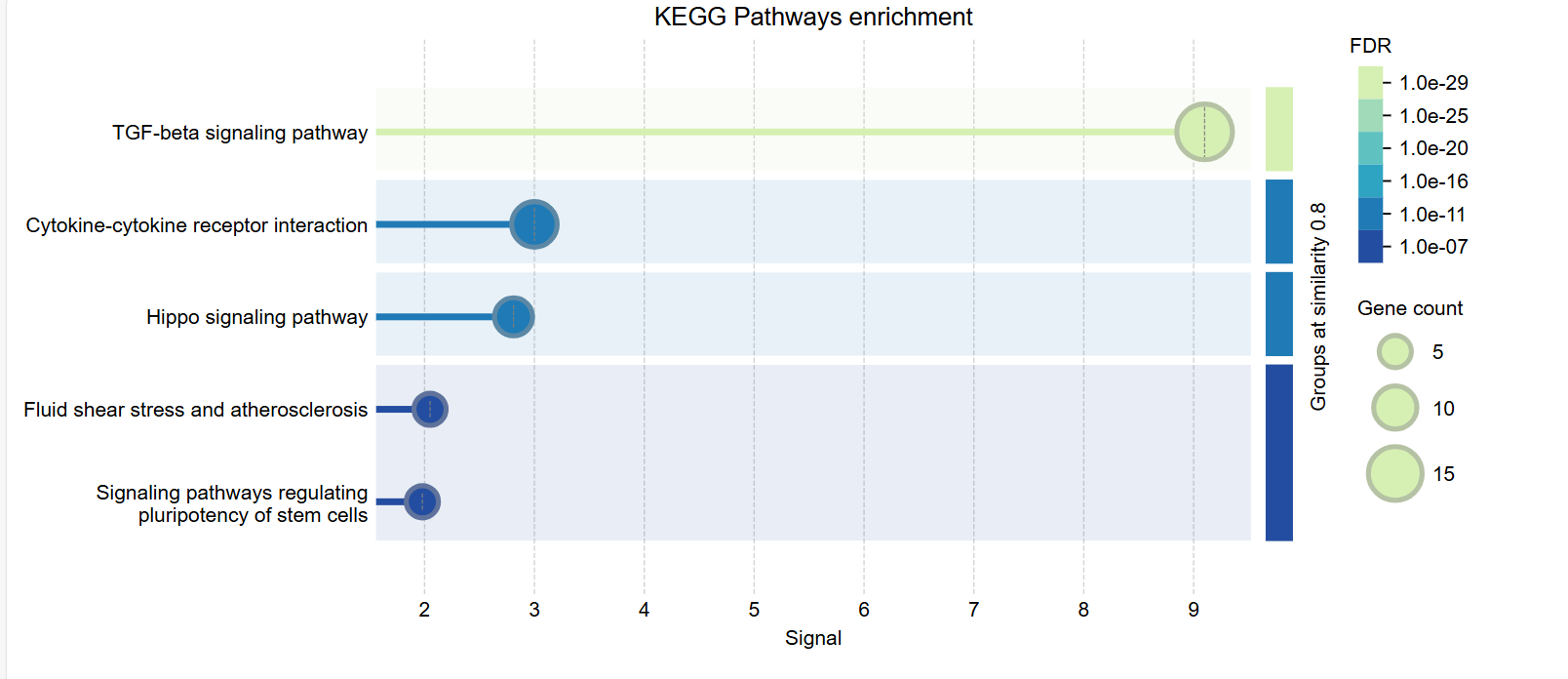

Supplement: Supplementary file 2 — Supplementary Material: brb371350‐sup‐0001‐SuppMat.doc [file BRB3-16-e71350-s001.doc]
