# Supplementary material for: The Mediating Effect of Inflammatory Biomarkers in the Associations Between Sarcoidosis and Incident Ischemic Stroke: A Prospective Cohort Study
Source: Brain Behav. 2026 Mar 26;16(4):e71350. doi: 10.1002/brb3.71350 (PMC13109034; doi:10.1002/brb3.71350)
Supplement: Supplementary file 3 — Supplementary Material: brb371350‐sup‐0001‐tableS1.doc [file BRB3-16-e71350-s003.doc]

### ****Supplementary Table S1. Baseline Characteristics of the Study Population After 1:4 Propensity Score Matching****

| CharacteristicsControl (n=7,040)Sarcoidosis (n=1,760)SMDP TrendSEX, n, (%)0.0080.786     Female3680 (52.3%)927 (52.7%)     Male3360 (47.7%)833 (47.3%)Age, years, mean (SD)56.72 (7.93)56.82 (7.80)0.0130.633Race, n, (%)0.0280.689     White6405 (91.0%)1606 (91.2%)     Other635 (9.0%)154 (8.8%)Health status, n, (%)0.0300.727     Excellent671 (9.5%)171 (9.7%)     Good3607 (51.2%)892 (50.7%)     Fair2175 (30.9%)536 (30.5%)     Poor587 (8.3%)161 (9.1%)Smoking status, n (%)0.0200.759     Never4474 (63.6%)1114 (63.3%)     Previous2228 (31.6%)554 (31.5%)     Current338 (4.8%)92 (5.2%)Drinking status, n (%)0.0060.978     Never415 (5.9%)102 (5.8%)     Previous317 (4.5%)78 (4.4%)     Current6308 (89.6%)1580 (89.8%)Education, n, (%)0.0330.956     College / University degree2405 (34.2%)593 (33.7%)     A /AS levels / equivalent822 (11.7%)214 (12.2%)     O levels/GCSEs / equivalent1454 (20.7%)377 (21.4%)     CSEs or equivalent406 (5.8%)98 (5.6%)     NVQ/HND/HNC / equivalent491 (7.0%)121 (6.9%)     Other professional qualifications442 (6.3%)101 (5.7%)     None of the above1020 (14.5%)256 (14.5%)Diabetes, n, (%)0.0060.858     NO6416 (91.1%)1607 (91.3%)     YES624 (8.9%)153 (8.7%)Hypertension, n, (%)0.0010.996     NO3659 (52.0%)914 (51.9%)     YES3381 (48.0%)846 (48.1%)Body mass index, kg/m², mean (SD)28.52 (5.34)28.56 (5.12)0.0070.785Townsend Deprivation Index, mean (SD)-1.10 (3.16)-1.15 (3.17)0.0180.504Triglycerides, mmol/L, mean (SD)1.85 (1.07)1.85 (1.01)0.0080.761Total cholesterol, mmol/L, mean (SD)5.61 (1.18)5.62 (1.14)0.0030.908HDL, mmol/L, mean (SD)1.37 (0.35)1.37 (0.37)0.0010.973LDL, mmol/L, mean (SD)3.55 (0.90)3.55 (0.86)0.0040.889 | |  | |  | |  | |  |
| --- | --- | --- | --- | --- | --- | --- | --- | --- |
|  |  | |  | |  | |  | |
|  |  | |  | |  | |  | |
|  |  | |  | |  | |  | |
|  |  | |  | |  | |  | |
|  |  | |  | |  | |  | |
|  |  | |  | |  | |  | |
|  |  | |  | |  | |  | |
|  |  | |  | |  | |  | |
|  |  | |  | |  | |  | |
|  |  | |  | |  | |  | |
|  |  | |  | |  | |  | |
|  |  | |  | |  | |  | |
|  |  | |  | |  | |  | |
|  |  | |  | |  | |  | |
|  |  | |  | |  | |  | |
|  |  | |  | |  | |  | |
|  |  | |  | |  | |  | |
|  |  | |  | |  | |  | |
|  |  | |  | |  | |  | |
|  |  | |  | |  | |  | |
|  |  | |  | |  | |  | |
|  |  | |  | |  | |  | |
|  |  | |  | |  | |  | |
|  |  | |  | |  | |  | |
|  |  | |  | |  | |  | |
|  |  | |  | |  | |  | |
|  |  | |  | |  | |  | |
|  |  | |  | |  | |  | |
|  |  | |  | |  | |  | |
|  |  | |  | |  | |  | |
|  |  | |  | |  | |  | |
|  |  | |  | |  | |  | |
|  |  | |  | |  | |  | |
|  |  | |  | |  | |  | |
|  |  | |  | |  | |  | |
|  |  | |  | |  | |  | |
|  |  | |  | |  | |  | |
|  |  | |  | |  | |  | |
|  |  | |  | |  | |  | |
|  |  | |  | |  | |  | |

*SMD, Standardized Mean Difference; An SMD < 0.1 indicates a negligible difference between groups and confirms successful balance after matching.
